# Insanity

**Published:** 1873-06

**Authors:** 


### INSANITY.

It ought to be deeply impressed on every intelligent mind, that of those who are sent to asylums for treatment and cure, within a year after their misfortune befalls them three-fourths are discharged cured, within a few months. If treatment is delayed beyond a year, the hope of restoration decreases with great rapidity; few ever leave their prison bars who have been insane for years. The great remedies for insanity are kindness and unrestraint in pleasant surroundings. Within less than a hundred years, to-day, in civilized, Christian America, the insane, in out of the way places, are treated as infuriated wild beasts, are chained to the floor or wall, and are kept subdued by stripes and blows from thongs and cudgels. At the date above there was in England a building, placed in a low confined situation, no windows in front, and every chink barred and grated; worse than a jail, for when you entered, above the creaks of hinges and the clanging of chains, arose the shrieks and sobs and execrations and wild sayings of the miserable inmates; poisonous emanations were floated through dark and damp and narrow passages. The keeper walks ahead of the visitor with a bunch of keys and a small whip, and speaks only in harsh and angry monosyllables. A door is opened admitting to a room, twelve by seven, with a small window which never opens; in this pen are ten women, with no other covering than a rag around the waist, loathsome and hideous; each chained to the wall. In sorrow or fear or shame, one utters a cry, which is instantly quieted with a blow on the temples from the fist of the unfeeling, savage keeper; blood from the head, and a tear from the eye, and a cower from the heart; another chain, a gag, and an indecent expression, and then comes silence. In a kennel, five of these women sleep in a space of eight feet square. No bedding but wet and musty and rotting straw, emitting a stench which drives the visitor hastily away.

One day in 1791 a lady, a member of the Society of Friends, was taken to the asylum. Her friends were not allowed to see her; she died. Wrong doing was suspected, and a number of the members of the same Society determined to have an institution where there should be no secrets; and incited thereto more particularly was

FRIEND WILLIAM TUKE, who visited St. Luke's hospital in London, one day, and saw a young woman, without clothing, chained to the wall, and lying on some loose straw, dirty and damp. This sight so haunted him, that he could not rest until he instituted measures which initiated a new asylum, of which he became president, located at York and called the "Retreat," with great thoughtfulness that the patients' minds might not be harassed with thoughts of jails and iron bars and clanking keys. "Cheerful and salutary amusements" were instituted; an effort was made to cherish in the patients the strengthening and consolatory principles of religion and virtue. Tea parties were given now and then, to which certain inmates were invited by the officers of the institution, as their friends. No ball and chain, no clogs, no handcuffs, were ever allowed to come inside the doors. Note the result. A patient was admitted who in another institution had been chained and naked for twenty years. In a short time he was induced to "wear clothing, and to adopt orderly habits."

Twenty years later, when this institution was visited, it was found that persons were sent there from all parts of England, frantic, ungovernable, and in chains. They were at once released, and by gentle treatment and easy arguments were sometimes in a few hours apparently "clothed and in their right minds."

Dr. Kirkbride, another member of the Society of Friends, is the superintendent of an Insane Asylum, at Philadelphia; for twenty or thirty years he has patiently, wisely, and laboriously been gradually extending this line of treatment, employing the patients in healthful exercises, in horticulture, in music, in paintings and engravings, the great instrumentalities being gentleness, encouragements, physical and mental occupations of an agreeable character. He loves his work, and in it has secured the confidence and respect and love of all who know him. What Friend Dorothea Dix has done in the direction of ameliorating the condition of the hapless insane, is known to intelligent readers, and her memory will be honored while her works will follow. Thus it is seen that to the Quakers belong the glory of inaugurating the better way for treating lunatics.

